# Kounis Syndrome: A Report of Two Cases and a Review of the Literature

**DOI:** 10.7759/cureus.96439

**Published:** 2025-11-09

**Authors:** Sergio Gomez-Olarte, Emiliano Zuleta-Housni, Luis Areiza

**Affiliations:** 1 Department of Interventional Cardiology, Hospital Universitario Mayor - Mederi, Bogota, COL

**Keywords:** allergy, coronary artery disease, hypersensitivity, kounis syndrome (ks), myocardial infarction, stent thrombosis

## Abstract

Kounis syndrome (KS) is a rare condition in which allergic reactions activate mast cells and platelets, causing acute coronary syndromes (ACS), such as myocardial infarction, coronary vasospasms, or stent thrombosis. It can be triggered by drugs, food, vaccines, or insect bites. We report two cases involving elderly male patients.

The first case describes a 71-year-old male with an allergy to non-steroidal anti-inflammatory drugs (NSAIDs), who accidentally ingested diclofenac and developed an allergic reaction with chest pain. In the emergency room, he was diagnosed with ST-elevation acute myocardial infarction (STEMI) and stent thrombosis in the right coronary artery, confirming a type III KS diagnosis. The second case involves a 73-year-old male with a history of prostate cancer and cerebral aneurysm, who had an allergic reaction after consuming dairy products, which was treated with cetirizine. Eight months later, he experienced a more severe reaction after eating fish, presenting with dizziness and chest pain. Elevated troponin levels and electrocardiographic changes indicated NSTEMI. Thereafter, it transitioned into a STEMI with atrial fibrillation with rapid ventricular response and was treated with thrombolytics and amiodarone. Finally, a medicated stent was implanted in the main vessel after a coronary arteriography.

These cases highlight the importance of considering allergic etiologies in the differential diagnosis of acute coronary syndromes to ensure accurate identification and tailored treatment.

## Introduction

Kounis syndrome (KS) is an acute coronary syndrome (ACS) precipitated by mast-cell activation in the setting of allergic, hypersensitivity, or anaphylactic reactions. First described by Kounis and Zavras in 1991, it has since been referred to as “allergic angina” or “allergic myocardial infarction” [1]. The release of histamine, leukotrienes, and other mediators induces coronary vasospasm, promotes platelet aggregation, and destabilizes atherosclerotic plaques. Histamine in particular exerts potent effects through H1- and H2-receptors distributed across cardiac chambers and coronary arteries, and clinical as well as experimental data confirm its role in provoking angina and myocardial infarction [1].

Although the true prevalence of KS remains uncertain, it is likely underdiagnosed worldwide. In the United States, a seven-year analysis of 253,420 allergic reactions estimated an annual prevalence of approximately 1.1% [2]. While local epidemiological data are scarce, individual cases related to inadvertent drug intake, food allergens, or insect stings have been reported. Beyond its frequency, KS carries significant clinical and economic implications. In a 2019 study by Desai et al., patients with KS demonstrated higher in-hospital all-cause mortality (odds ratio (OR) 9.74; 95% confidence interval (CI): 8.08-11.76; p<0.001), longer hospital stays (mean: 5.8 ± 6.0 vs. 3.0 ± 3.9 days; p<0.001), and greater hospitalization costs (USD 52,656 vs. USD 20,487; p<0.001) compared with patients without KS [3]. These findings highlight the urgent need for heightened awareness and systematic recognition of KS.

KS has been described across all races, age groups (9-90 years), and geographic regions, with the highest incidence occurring between 40 and 70 years of age (68%) [4]. Established risk factors include prior allergy, hypertension, smoking, diabetes, and hyperlipidemia. The spectrum of triggers continues to expand, with antibiotics (27.4%) and insect bites (23.4%) being the most frequently implicated, although many triggers remain unidentified [4]. Diagnosis is primarily based on clinical suspicion, which contributes to its frequent underrecognition in emergency settings.

Three clinical variants of KS have been described. Type I involves coronary artery spasm in patients with normal or near-normal coronary arteries and no traditional risk factors for coronary artery disease. This form is thought to reflect endothelial dysfunction or microvascular angina. In these cases, the acute release of inflammatory mediators can induce transient coronary spasm, which may occur without elevation of cardiac enzymes or, in more severe cases, progress to myocardial infarction with biomarker elevation [5]. Type II occurs in patients with underlying, often asymptomatic, atherosclerotic disease. Here, the allergic response may precipitate coronary spasm in conjunction with plaque rupture or erosion, resulting in an acute myocardial infarction [5]. Type III is observed in patients with prior percutaneous coronary interventions (PCIs), where inflammatory mediators provoke stent thrombosis. Histological analysis of thrombus aspirates in these cases typically reveals eosinophils and mast cells when stained with hematoxylin-eosin and Giemsa, respectively [5].

Despite growing awareness, KS remains systematically underdiagnosed, and no standardized diagnostic or therapeutic algorithms currently exist. Continued reporting of atypical presentations and severe phenotypes is therefore essential to enhance clinical awareness, refine diagnostic approaches, and guide the development of evidence-based management strategies.

## Case presentation

Case 1

A 71-year-old male with a medical history of hypertension, diabetes mellitus, rheumatoid arthritis, right-sided Bell’s palsy, and ischemic cardiomyopathy secondary to coronary artery disease with prior stent placement (anatomy unknown) presented to the emergency department with acute-onset, severe, non-radiating chest pain of one-hour duration, consistent with cardiac-type pain. The symptoms developed immediately following inadvertent ingestion of diclofenac, a nonsteroidal anti-inflammatory drug (NSAID) to which he had a known allergy.

On arrival, ECG revealed ST-segment elevation in the inferior leads (DII, DIII, and aVF), accompanied by bradycardia, type I hypertension, and first-degree atrioventricular block. The patient was treated for his allergic reaction with an antihistamine and corticosteroid. Given the high suspicion of ACS, an urgent interventional cardiology consultation was obtained, and he was transferred to the catheterization laboratory for emergent PCI after receiving a 300 mg loading dose of aspirin. Coronary angiography confirmed the diagnosis of ST-segment elevation myocardial infarction (STEMI), and early PCI was successfully performed.

**Figure 1:**
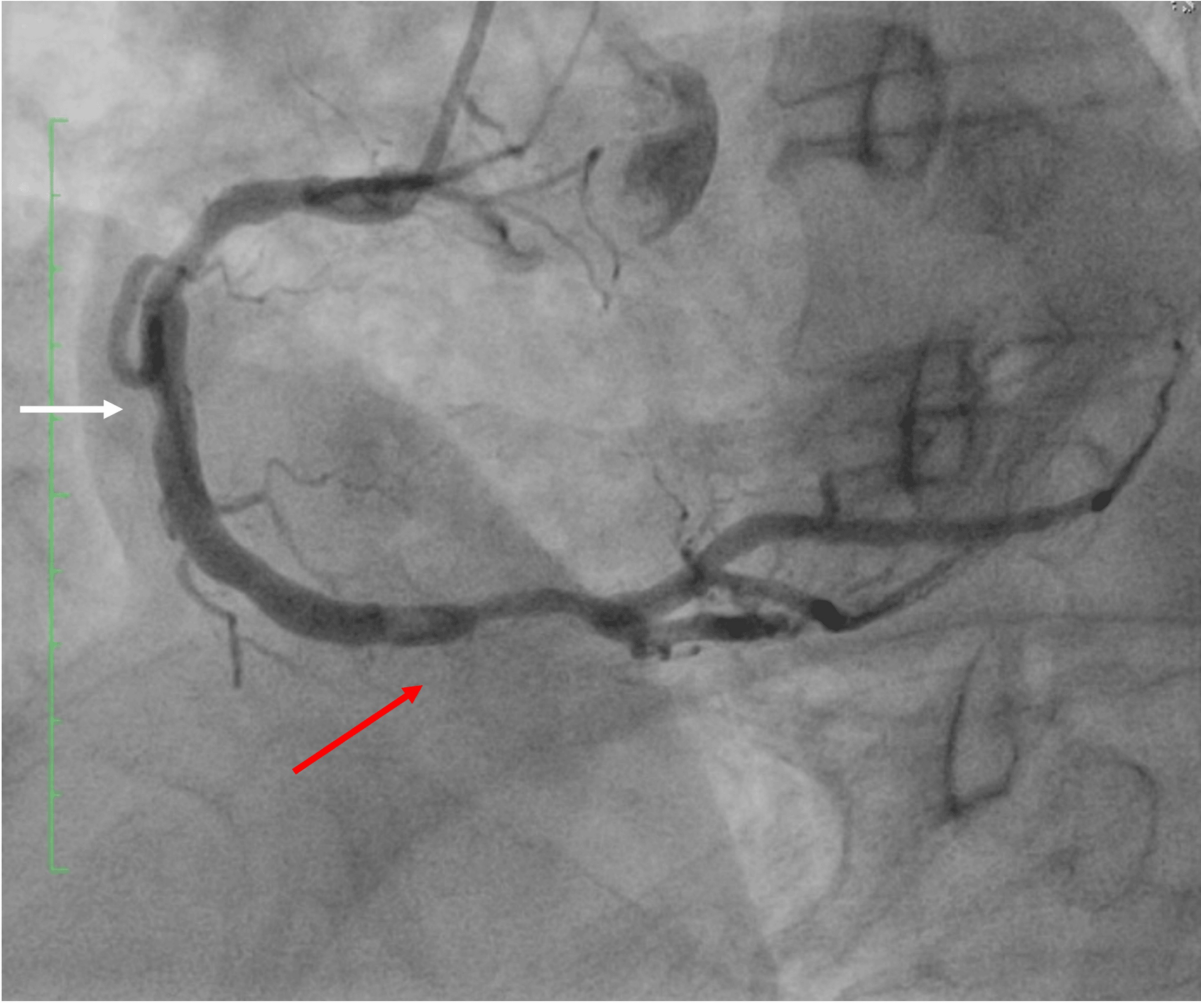
Diagnostic coronary arteriography Fluoroscopy showing a 70% lesion in the proximal third (white arrow) and stent restenosis in the distal third of the right coronary artery (red arrow)

Coronary angiography revealed a 70% proximal lesion and in-stent thrombosis occluding 50% of the right coronary artery (RCA) lumen, consistent with a diagnosis of type III KS (Figure 1).

**Figure 2:**
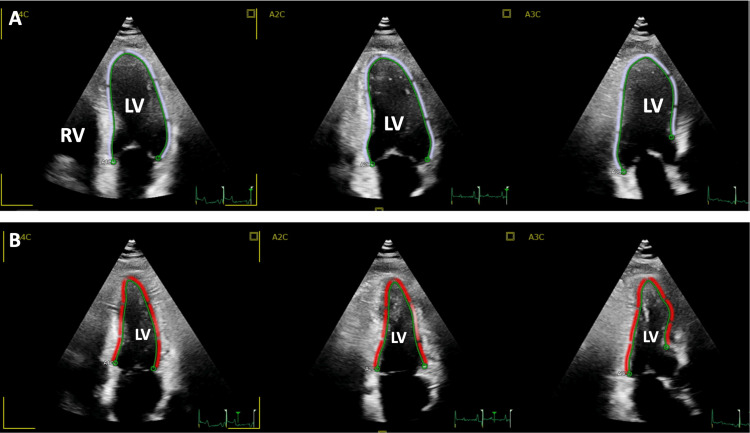
Apical echocardiographic views showing normal left ventricular function Echocardiographic apical four-chamber views at end-diastole (A) and end-systole (B) demonstrate normal wall motion of the LV without evidence of contractility or valvular abnormalities. LV: left ventricle; RV: right ventricle

The patient was started on an 18-hour intravenous infusion of the glycoprotein IIb/IIIa inhibitor tirofiban, and percutaneous transluminal coronary angioplasty was scheduled for a second-stage angioplasty. Transthoracic echocardiography (TTE) was performed, showing no wall motion abnormalities or valvular disease, with a preserved left ventricular ejection fraction (LVEF) of 67% (Figure 2).

**Figure 3:**
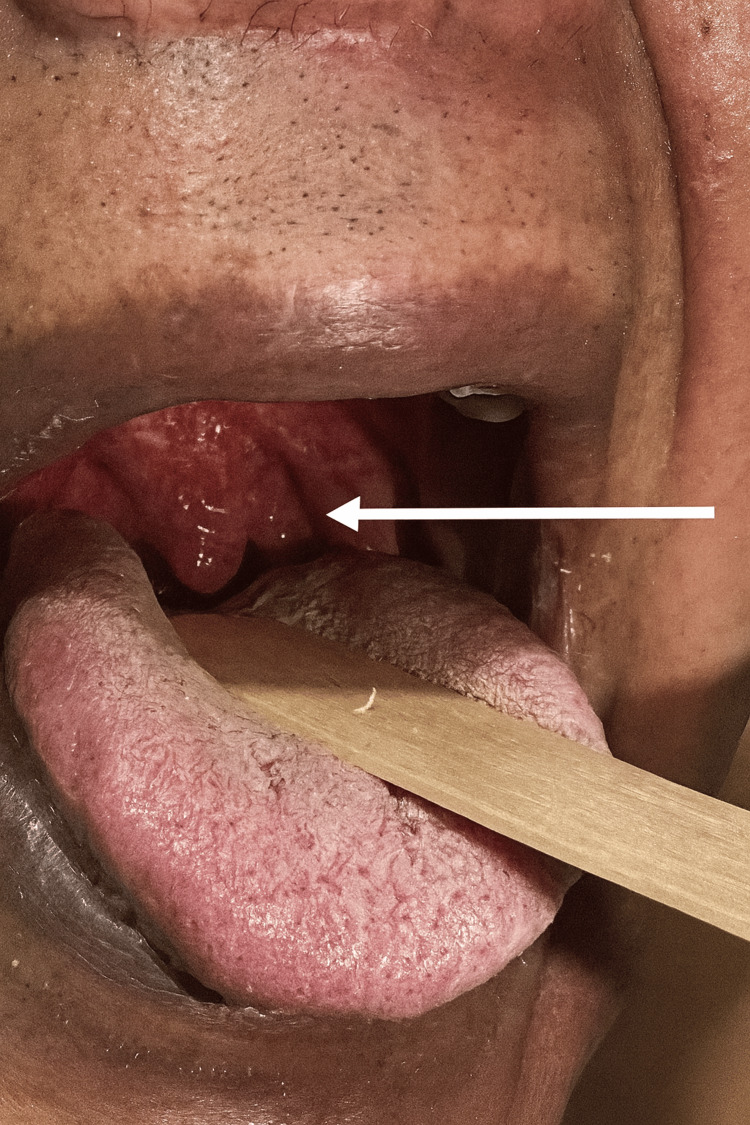
Inflammation of the uvula due to an inflammatory process associated with type III Kounis syndrome Oropharyngeal examination showed marked erythema and swelling of the uvula (white arrow), consistent with uvulitis. This finding was part of a systemic hypersensitivity reaction compatible with type III Kounis syndrome

During recovery, uvular and oropharyngeal edema was noted; otolaryngology evaluation confirmed allergic etiology without airway compromise, and IV antihistamines and corticosteroids were continued (Figure 3).

**Figure 4:**
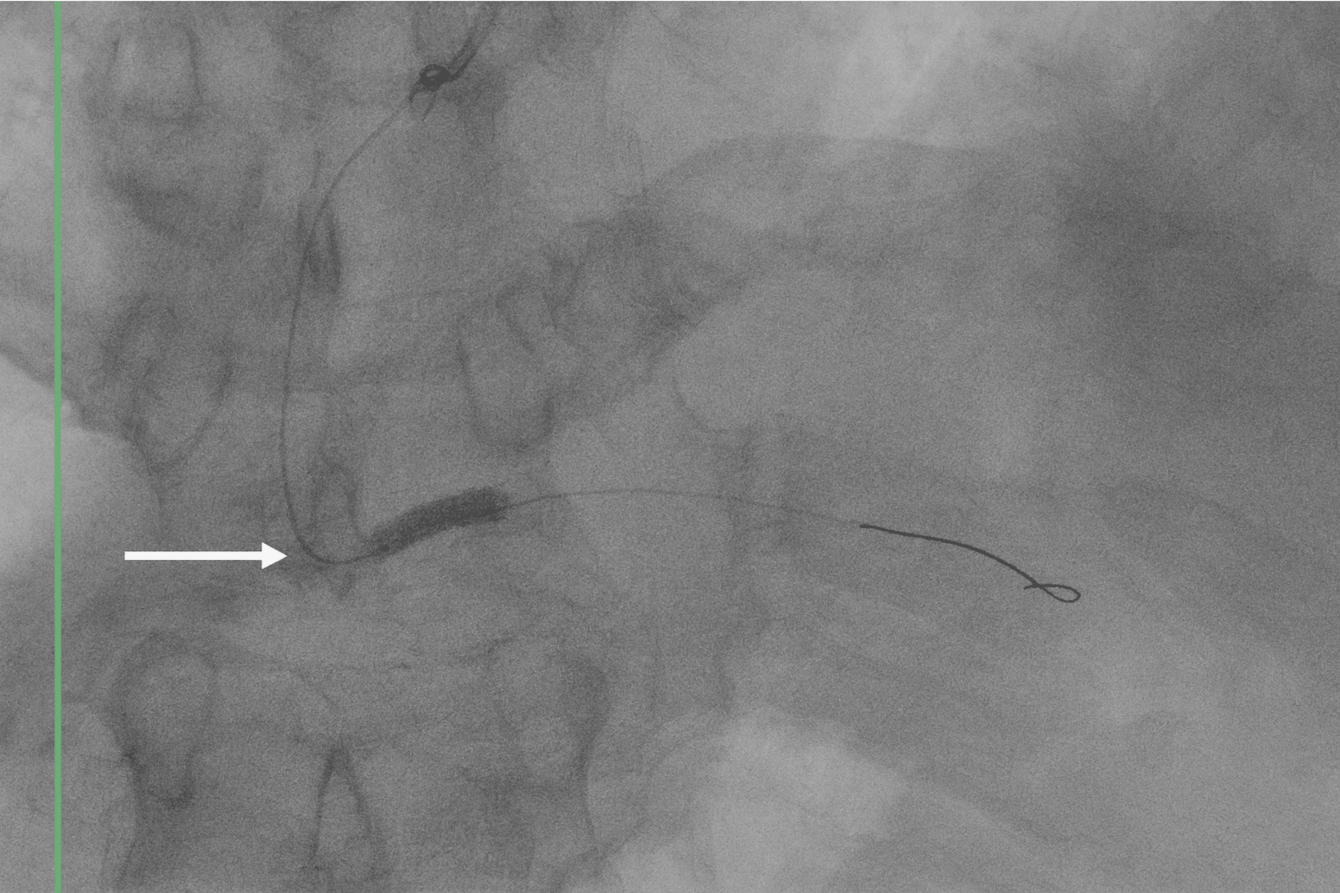
Percutaneous transluminal coronary angioplasty (PTCA) showing balloon dilation of restenosis in the distal segment of the right coronary artery Fluoroscopic image demonstrating balloon inflation during percutaneous transluminal coronary angioplasty (PTCA) for the treatment of in-stent restenosis in the distal third of the right coronary artery (white arrow)

**Figure 5:**
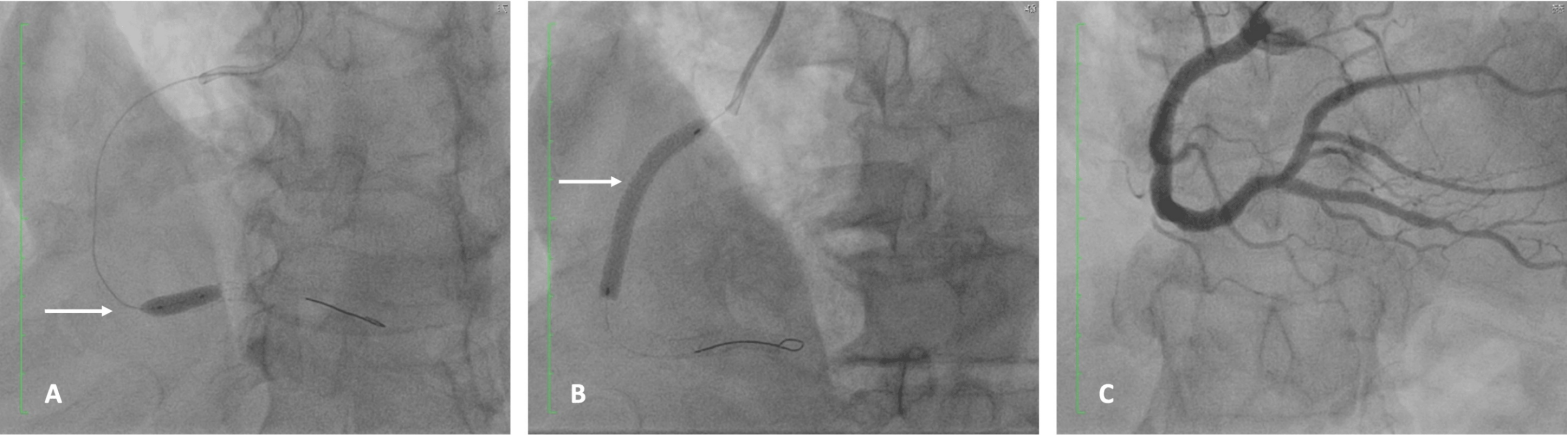
Stepwise percutaneous coronary intervention of the RCA (A) Angioplasty with a 3.5 × 20 mm drug-coated balloon (Agent) for the treatment of distal in-stent restenosis of the right coronary artery (white arrow). (B) Subsequent angioplasty followed by implantation of a drug-eluting stent in the mid-segment of the RCA (white arrow). (C) Final angiographic result demonstrating optimal vessel expansion and restoration of coronary flow RCA: right coronary artery

After completing the tirofiban infusion, a second-stage PCI was performed. The procedure included balloon angioplasty of the right coronary artery using a drug-coated balloon to treat in-stent restenosis (Figure 4), followed by implantation of a drug-eluting stent in the mid-RCA (Figures 5A, 5B, 5C).

The patient was subsequently transferred to the cardiology ward, where his clinical condition remained stable. On the third day of hospitalization, he was discharged with complete resolution of symptoms and scheduled for follow-up in the interventional cardiology clinic.

Case 2

A 73-year-old male with a history of prostate cancer treated with prostatectomy, cerebral aneurysm clipping in 2010, and a dental abscess on amoxicillin/clavulanate presented to the emergency department with recurrent allergic symptoms. He had previously experienced facial edema and pruritus after consuming dairy products, for which he received a single 50 mg oral dose of prednisone and was discharged on cetirizine 10 mg once daily for seven days. On this occasion, he presented again to the emergency department with a similar episode of approximately four hours’ duration after consuming tilapia, papaya, borojó, and his prescribed antibiotic. The episode was accompanied by dizziness, headache, dyspnea, generalized edema, and pruritus. On admission, the patient received a single IV dose of clemastine (2 mg/2 mL) and metoclopramide (10 mg). During evaluation, he reported non-cardiac-type chest pain. A 12-lead ECG revealed J-point elevation in the inferior leads, particularly in lead aVF, along with positive troponin levels. He was subsequently transferred to the coronary care unit (CCU) for management of a non-ST-segment elevation myocardial infarction (NSTEMI). TTE demonstrated regional wall motion abnormalities with a preserved LVEF of 53%.

**Figure 6:**
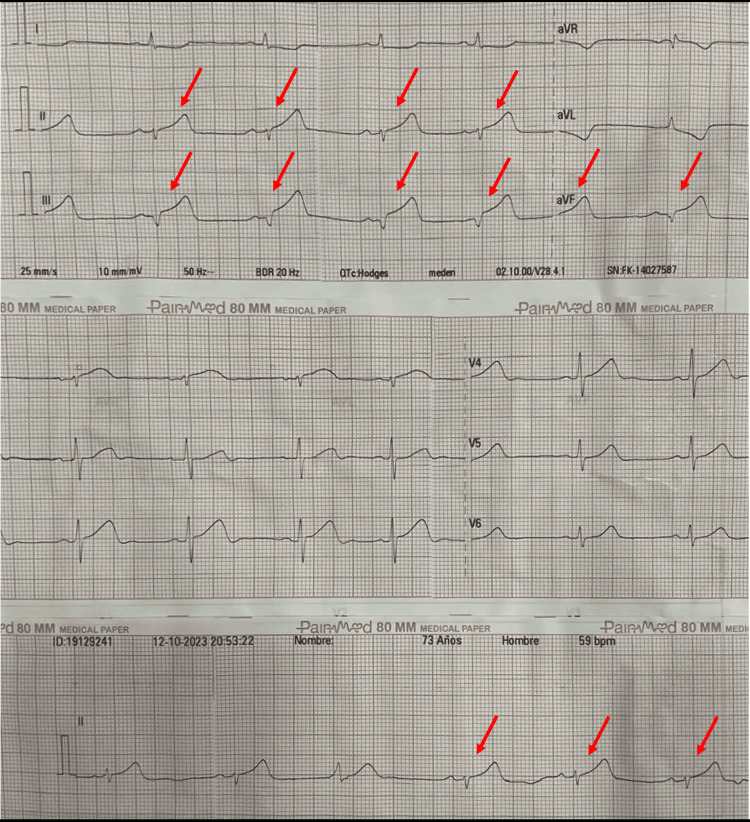
ECG following symptom recurrence showing inferior ST-segment elevations Twelve-lead ECG showing sinus rhythm with ST-segment elevation in the inferior leads (II, III, aVF) indicated by red arrows ECG: electrocardiogram

The patient was evaluated by the interventional cardiology team, who noted no angina-equivalent symptoms and improvement of chest pain. He was considered to have possible ischemic heart disease with stable coronary artery disease, and outpatient invasive coronary stratification was recommended. Later, the patient developed diaphoresis and severe precordial chest pain radiating to the left upper limb and cervical region, rated 8 out of 10 in intensity. An ECG revealed an inferior STEMI involving leads DII, DIII, and aVF (Figure 6).

Thrombolytic therapy was initiated with 70 mg of IV alteplase (0.9 mg/kg for a body weight of 70 kg), consisting of a 7 mg bolus followed by 63 mg infused over 60 minutes. Following coordination with the interventional cardiology service, adjunctive therapy was initiated, including 30 mg IV enoxaparin followed by 70 mg subcutaneously, along with loading doses of 300 mg of aspirin and 300 mg of clopidogrel administered orally. Continuous clinical monitoring was maintained. Three hours later, the patient developed grade II hypertension (150/122 mmHg) but reported improvement in chest pain, rated 4 out of 10 on the visual analog scale (VAS). A titratable IV nitroglycerin infusion was started at 0.5 μg/kg/min according to clinical response. One hour later, cardiac monitoring revealed new-onset atrial fibrillation with rapid ventricular response, without hemodynamic compromise or recurrent chest pain. A 150 mg IV bolus of amiodarone was administered, successfully restoring sinus rhythm.

**Figure 7:**
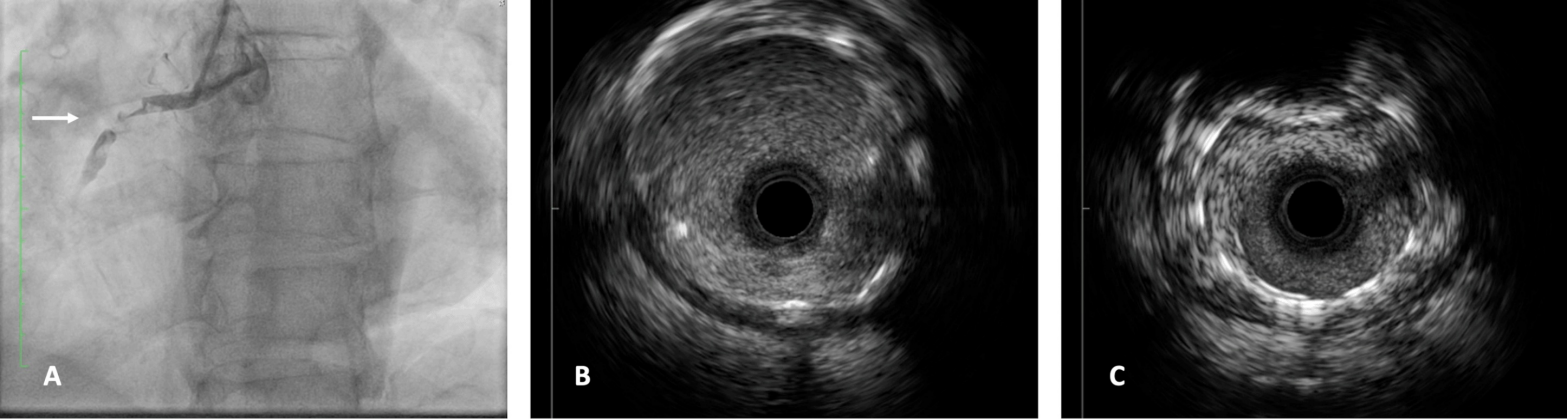
Diagnostic coronary arteriography and IVUS findings of the RCA (A) Diagnostic coronary arteriography: fluoroscopy showing a lesion in the proximal third of the right coronary artery (white arrow). (B) IVUS demonstrating stent restenosis in the distal third of the RCA. (C) IVUS image revealing thrombus formation in the proximal segment of the RCA IVUS: intravascular ultrasound; RCA: right coronary artery

Coronary angiography was performed, which revealed a myocardial bridge in the left anterior descending artery causing approximately 30% systolic luminal narrowing with a good distal bed; the first obtuse marginal artery presented a 20% proximal plaque and a chronic total occlusion at the ostial level; and the right coronary artery (dominant vessel) showed an ulcerated proximal lesion with a large thrombus burden, distal TIMI (thrombolysis in myocardial infarction) I flow (penetration without perfusion), and an adequate distal bed (Figures 7A, 7B, 7C).

**Figure 8:**
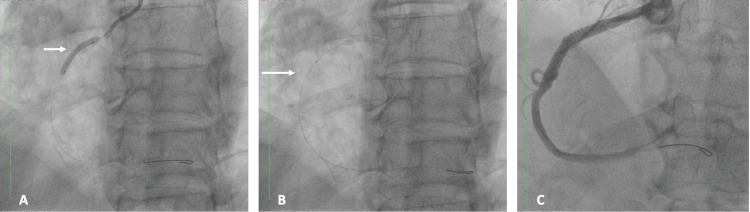
Stepwise PCI of the RCA (A) Angioplasty of the RCA (white arrow). (B) Thrombus aspiration of the RCA (white arrow). (C) Final angiographic result following successful angioplasty, showing restored vessel patency and normal distal flow PCI: percutaneous coronary intervention; RCA: right coronary artery

The findings were consistent with severe coronary artery disease involving one major and one secondary vessel, with a high suspicion of KS. Following the diagnostic catheterization, PCI was performed, including thromboaspiration and deployment of a drug-eluting stent in the right coronary artery (Figures 8A, 8B, 8C).

A serum tryptase assay was conducted, yielding a negative result. Histopathological examination of the thrombus aspirated during catheterization revealed microscopic evidence of degranulating cells, which stained positively with Giemsa, consistent with mast cells.

Both patients experienced a favorable clinical course with complete recovery and no adverse events. They were discharged within the first week of hospitalization in stable condition. At follow-up in the interventional cardiology clinic, the postoperative course and procedural outcomes were satisfactory. Dual antiplatelet therapy with clopidogrel was maintained for at least one year, and anticoagulation was continued under the guidance of the cardiology service.

## Discussion

Kounis syndrome, although traditionally considered uncommon, is likely underdiagnosed rather than truly rare. It represents a clinically significant intersection between allergic reactions and acute coronary events. Epidemiological evidence suggests that KS affects approximately 1.1% of patients hospitalized for allergic, hypersensitive, or anaphylactic episodes in the United States, with a mortality rate of around 7% [3]. Recent data from a comprehensive review by Yakushin et al. [6], which analyzed 235 clinical cases published over the last 32 years, further support this underrecognized burden. In that study, type I KS accounted for 49.7% of cases, type II for 27.2%, and type III for only 5.9%. The most frequent precipitants were antibiotics (32.3%) and NSAIDs (24.3%), while food allergens represented an additional but relevant subgroup. These findings closely align with our observations: both of our patients presented with two of the leading triggers identified in the review - NSAIDs and food allergens - reinforcing their central etiological role in KS worldwide.

Moreover, Yakushin et al. reported a median patient age of 57 years and a predominance of male patients (68.5%), consistent with the demographic profile of our cases. In their analysis, chest pain was the most common presenting symptom (59.1%), and ST-segment elevation was observed in 42.9% of patients-findings that mirror the clinical presentation of our cases, one with NSTEMI progressing to STEMI and another with inferior STEMI. These similarities underscore that, despite its apparent rarity, KS exhibits reproducible clinical and electrocardiographic patterns that may facilitate early recognition.

The triggers in our patients - diclofenac and food allergens - align with the most commonly reported precipitants in the literature, which include drugs, insect stings, and foods [5-7]. Notably, our first case provides rare in vivo evidence of stent thrombosis secondary to NSAID exposure, corresponding to type III KS: a category identified in only 5.9% of cases in the systematic review by Yakushin et al. [6]. This finding underscores the diagnostic and therapeutic complexity of allergic thrombosis superimposed on pre-existing coronary disease. Conversely, our second case, involving rapid progression from NSTEMI to STEMI during a food-induced allergic reaction, illustrates the dynamic spectrum of coronary involvement and supports the concept of accelerated thrombus formation under allergic inflammation. Together, these uncommon but clinically relevant scenarios expand upon existing evidence, emphasizing that KS may manifest not only as coronary vasospasm but also as abrupt thrombotic occlusion that closely mimics conventional ACS.

From a pathophysiological standpoint, the mechanism involves mast-cell degranulation with the release of histamine, leukotrienes, tryptase, platelet-activating factor, and cytokines, which can induce vasospasm, promote platelet aggregation, and destabilize atherosclerotic plaques [5,8]. Our first case provides rare in vivo evidence of thrombosis at a previously revascularized site, supporting the notion that allergic cascades can directly precipitate stent failure. Conversely, the second case illustrates how allergic inflammation can rapidly shift the clinical spectrum from vasospasm to full-thickness infarction. This underscores the wide spectrum of stimuli capable of activating mast cells and inducing coronary vasospasm or thrombosis.

KS remains a diagnostic challenge [2-9]. Both of our patients initially presented with allergic manifestations and only later developed chest pain and electrocardiographic changes, a sequence that often leads to underrecognition and misclassification as primary ACS. Clinicians should consider this syndrome in patients presenting with acute coronary findings temporally associated with allergic symptoms such as urticaria, angioedema, bronchospasm, or anaphylaxis. A structured diagnostic approach that integrates the timing of allergen exposure with ECG changes, biomarker trends, and imaging findings is essential. Although serum tryptase measurement may support the diagnosis, its short half-life (approximately 90 minutes) and limited availability reduce its sensitivity [7]. Consequently, maintaining a high index of clinical suspicion remains the cornerstone of diagnosis.

Therapeutic management is equally complex. Current literature suggests that type I KS may resolve with antiallergic therapy alone, whereas types II and III often require simultaneous treatment of both the acute coronary syndrome and the allergic reaction [2,7,10]. However, there is no standardized approach, and common interventions may worsen outcomes. In our patients, treatment decisions required a multidisciplinary approach involving cardiology, immunology, and critical care, underscoring the need for integrated management strategies and highlighting the absence of robust evidence-based guidelines [11].

Two critical gaps emerge when our findings are compared with existing literature: (1) the lack of universal diagnostic criteria capable of distinguishing KS from conventional ACS, and (2) the absence of therapeutic algorithms that integrate interventional cardiology with allergy and immunology principles. These challenges are compounded by the therapeutic paradox: treatments for the allergic reaction may exacerbate myocardial injury - such as H1-antihistamines causing hypotension and coronary hypoperfusion, or epinephrine increasing myocardial oxygen demand - while ACS-directed therapies do not address the underlying anaphylactic process [12]. Overcoming these barriers requires multicenter registries and collaborative research aimed at developing validated, multidisciplinary strategies for diagnosis and management.

Ultimately, the successful treatment of Kounis syndrome depends on heightened awareness, early recognition, and prompt intervention by first responders and clinicians. A timely, integrated approach that balances the management of both the allergic and cardiovascular components is essential to improving outcomes in this complex and potentially life-threatening condition.

## Conclusions

KS is not a rare entity but rather a substantially underdiagnosed condition due to its broad and evolving spectrum of clinical manifestations and triggers. It should be considered early in the differential diagnosis of chest pain, given its potential to transform an apparently benign allergic reaction into a life-threatening myocardial infarction or even sudden cardiac death. Clinicians must remain vigilant for its diverse precipitants - including insect stings, medications, contrast agents, foods, and other substances capable of inducing mast-cell activation. Our cases illustrate rare but clinically meaningful presentations - NSAID-induced stent thrombosis and rapid progression from NSTEMI to STEMI - that broaden the recognized spectrum of KS. The key message is clear: integrating awareness of KS into routine cardiovascular assessment is essential, as timely recognition and tailored management can be decisive in improving patient outcomes.
